# Alopecia and Semaglutide: Connecting the Dots for Patient Safety

**DOI:** 10.1111/jocd.70125

**Published:** 2025-03-15

**Authors:** Diala Haykal

**Affiliations:** ^1^ Centre Laser Palaiseau, private practice Palaiseau France

**Keywords:** alopecia, Glucagon‐like peptide‐1 (GLP‐1) receptor agonist, hairloss, semaglutide

To the Editor,

Semaglutide, a glucagon‐like peptide‐1 (GLP‐1) receptor agonist, has rapidly gained popularity in clinical practice due to its remarkable efficacy in controlling blood glucose levels in patients with type 2 diabetes mellitus and promoting significant weight loss [1]. Despite these clear clinical benefits, emerging evidence has raised concerns about its potential dermatological adverse effects, specifically hair loss or alopecia, warranting further detailed examination. Recent studies have begun shedding light on this potentially overlooked side effect. Burke et al. conducted a pivotal retrospective cohort study, uncovering a notable association between the use of GLP‐1 receptor agonists, including semaglutide, and an increased incidence of alopecia. Their findings underline the critical need for prospective, randomized controlled studies to validate and quantify the strength of this association accurately [2]. Complementing these findings, a scoping review by Tran et al. systematically documented a range of dermatologic complications associated with semaglutide, including frequent reports of alopecia. This comprehensive review underscores the necessity for clinicians to maintain heightened vigilance when prescribing this medication, as hair loss can have profound implications for patient well‐being and treatment adherence [3]. Additionally, Godfrey et al. provided significant contributions through a disproportionality analysis of data from the FDA Adverse Event Reporting System (FAERS). Their analysis reported notable associations between the use of semaglutide and another GLP‐1 agonist, tirzepatide, with increased reporting of alopecia. Although causality cannot be definitively established through FAERS, the consistency across independent reports enhances the biological plausibility of a connection between semaglutide and hair loss [4]. Further emphasizing the importance of structured clinical research, Desai et al. highlighted an urgent need for more comprehensive prospective studies focused explicitly on this phenomenon. The authors call attention to the limitations of current evidence, which largely originates from observational data, stressing the critical importance of rigorous scientific inquiries to better define risk profiles associated with GLP‐1 receptor agonists [5].

Potential mechanisms linking semaglutide use to alopecia deserve further investigation. The rapid and substantial weight reduction associated with semaglutide therapy can precipitate nutritional deficiencies, particularly of micronutrients like iron, zinc, vitamin D, and biotin. These deficiencies are well‐recognized triggers for telogen effluvium, a type of diffuse hair shedding resulting from disruption in the hair growth cycle. Furthermore, endocrine disruptions, especially thyroid hormone fluctuations due to GLP‐1 receptor agonists, could adversely affect hair follicle cycling. Given the essential role of thyroid hormones in maintaining healthy hair follicle function, even subtle hormonal imbalances could significantly contribute to hair loss [6]. Moreover, the psychosocial consequences of alopecia associated with semaglutide treatment must not be overlooked. Hair loss often carries significant emotional distress, negatively affecting self‐esteem, mental health, and overall quality of life. Addressing these psychological impacts is crucial, as they can profoundly influence patient satisfaction, adherence to treatment, and overall therapeutic outcomes [1, 7]. Therefore, clinicians must adopt proactive, interdisciplinary approaches, including early identification of alopecia, nutritional interventions, psychological counseling, and dermatologic consultations.

In conclusion, although preliminary evidence strongly suggests an association between semaglutide therapy and alopecia, definitive proof remains lacking. Comprehensive and robust clinical trials focusing explicitly on hair health, scalp conditions, nutritional status, hormonal regulation, and psychosocial outcomes are urgently needed. Such dedicated research endeavors will facilitate safer clinical practices and optimized patient care strategies, ensuring maximum therapeutic benefit while minimizing adverse effects.

## Conflicts of Interest

The author declares no conflicts of interest.

## Data Availability

The data that support the findings of this study are available in the references' part.
